# KRAS-driven cytokine–metabolic crosstalk shapes immune exclusion in pancreatic ductal adenocarcinoma

**DOI:** 10.3389/fgene.2026.1899014

**Published:** 2026-08-24

**Authors:** Chengjun Huang, Gang Mai, Yu Li, Hang Liu, Wei Huang

**Affiliations:** 1 Department of Pancreatitis Treatment Center, People’s Hospital of Deyang City, Deyang, China; 2 Department of Hepatobiliary Surgery, People’s Hospital of Deyang City, Deyang, China; 3 Department of Gastrointestinal Surgery, People’s Hospital of Deyang City, Deyang, China

**Keywords:** cytokine signaling, immune exclusion, KRAS, metabolic reprogramming, pancreatic ductal adenocarcinoma, tumor microenvironment

## Abstract

Pancreatic ductal adenocarcinoma (PDAC) remains refractory to immunotherapy, frequently characterized by an immune-excluded tumor microenvironment (TME). Oncogenic KRAS mutation, the predominant genetic driver in PDAC, serves as a master upstream regulator linking tumor-intrinsic signaling with microenvironmental remodeling. Through reshaping cytokine secretion and metabolic programs, mutant KRAS coordinates inflammatory and metabolic networks within the TME. Specifically, KRAS-linked cytokine networks, including tumor-cell-intrinsic mediators such as GM-CSF and microenvironment-associated inflammatory signals involving CXCLs, IL-6, TGF-β, and IL-1β, interact reciprocally with metabolic alterations, such as glycolytic activation, lactate accumulation, hypoxia adaptation, adenosine signaling, and altered amino acid and lipid metabolism. These pathways converge on shared regulatory nodes, notably STAT3, NF-κB, HIF-1α, and MYC, to drive immunosuppressive myeloid cell accumulation, stromal cell activation, extracellular matrix deposition, and restricted CD8^+^ T-cell infiltration. Furthermore, lactate, adenosine, and physical stromal barriers reinforce these immunosuppressive cascades, thereby establishing spatial immune exclusion. This review summarizes recent evidence on KRAS-driven cytokine–metabolic crosstalk in PDAC and discusses how this genotype-associated regulatory pattern governs immune exclusion, offering novel insights into overcoming immunotherapy resistance.

## 1 Introduction

Pancreatic ductal adenocarcinoma (PDAC) ranks as one of the deadliest solid tumors, with a 5-year survival rate of approximately 13%. Although immune checkpoint inhibitors have produced substantial clinical benefits in several malignancies, their efficacy in PDAC remains limited. PDAC exhibits heterogeneous immune states, including immune-desert, immune-excluded, and inflamed phenotypes (Chen and Mellman, 2017; Su et al., 2023). Most PDAC tumors display immune-desert or immune-excluded characteristics, with limited immune-cell infiltration or impaired penetration of effector T cells into tumor regions, whereas a smaller subset shows an inflamed phenotype with more abundant lymphocytic infiltration (Feig et al., 2013; de Santiago et al., 2019; Hegde and Chen, 2020). Among these immune states, immune exclusion represents a major barrier to effective antitumor immunity and immunotherapy response in PDAC. In this state, effector T cells fail to efficiently infiltrate the tumor parenchyma, which limits their direct contact with tumor cells and weakens antitumor immune responses. Therefore, immune exclusion is considered a major barrier to effective immunotherapy in PDAC, but the mechanisms underlying its development and persistence remain incompletely understood (Ho et al., 2020).

Cytokines and chemokines are important components of the immunosuppressive microenvironment in PDAC. Tumor cells promote the recruitment and expansion of myeloid-derived suppressor cells (MDSCs) and tumor-associated macrophages (TAMs) through the secretion of cytokines and chemokines, including interleukin-6 (IL-6), granulocyte–macrophage colony-stimulating factor (GM-CSF), and members of the CXC chemokine ligand (CXCL) family. These changes suppress effector T-cell function. Cancer-associated fibroblasts (CAFs) further reinforce this immunosuppressive state through interactions with immune cells (Mao et al., 2021). Recent single-cell studies have defined distinct CAF subsets in PDAC, including inflammatory CAFs (iCAFs), myofibroblastic CAFs (myCAFs), and antigen-presenting CAFs (apCAFs). These subsets may promote immune exclusion through cytokine secretion, extracellular matrix remodeling, and immune regulation. Several signaling axes have also been linked to immune exclusion. For example, CXCL1-driven neutrophil–tumor necrosis factor (TNF) signaling sustains inflammation and immunosuppression (Bianchi et al., 2023), whereas the CD200 pathway enhances the immunosuppressive microenvironment by regulating MDSC expansion (Choueiry et al., 2020). However, these studies have often discussed PDAC-associated immunosuppression mainly from the perspective of cytokine and inflammatory signaling, while its connection with metabolic changes has received less integrated attention.

Kirsten rat sarcoma viral oncogene homolog (KRAS) mutation is one of the most fundamental molecular features of PDAC and represents the predominant driver event in this disease, occurring in more than 90% of cases. Beyond promoting tumor cell proliferation and survival, oncogenic KRAS can reshape the secretory profile and metabolic state of tumor cells through downstream pathways such as mitogen-activated protein kinase (MAPK), phosphoinositide 3-kinase–AKT (PI3K–AKT), and signal transducer and activator of transcription (STAT) signaling. Previous studies have shown that KRAS mutation enhances tumor-cell responsiveness to microenvironment-derived cytokines, such as interleukin-4 (IL-4) and interleukin-13 (IL-13), by upregulating IL-2Rγ/IL-4Rα and IL-2Rγ/IL-13Rα1 receptor complexes. These cytokine signals activate the JAK1–STAT6–MYC pathway and promote glycolytic reprogramming in KRAS-mutant PDAC cells (Dey et al., 2020). KRAS-related signaling can also promote the recruitment of MDSCs and enhance immunosuppression through the nuclear factor-κB (NF-κB)–CXCL axis (Liu et al., 2023). However, not all inflammatory and metabolic changes in PDAC are directly driven by tumor-intrinsic KRAS signaling. Some inflammatory signals originate from stromal and immune cells, whereas metabolic alterations may also be shaped by hypoxia, nutrient availability, and other microenvironmental factors.

Metabolic reprogramming is another important layer of immune regulation in PDAC. Enhanced glycolysis and lactate accumulation can suppress T-cell activity by acidifying the TME. Altered glutamine and lipid metabolism may also weaken antitumor immunity through nutrient competition and signaling regulation. Lactate is not merely a metabolic byproduct. It can also regulate immune cell function and cytokine expression, thereby promoting an immunosuppressive state and reducing sensitivity to immunotherapy (Sun et al., 2025). In addition, metabolic pathways such as nucleotide metabolism have been implicated in tumor immune regulation (Liu Q et al., 2025). These findings indicate that metabolic alterations may directly participate in immune suppression, rather than simply accompany tumor progression.

Previous studies have identified several mechanisms of immune suppression in PDAC. IL-6, GM-CSF, and CXCL family members promote myeloid-cell recruitment and inflammatory signaling, whereas enhanced glycolysis, lactate accumulation, acidic TME formation, and adenosine signaling impair T-cell activity. CAF activation and extracellular matrix (ECM) deposition further restrict CD8^+^ T-cell infiltration into the tumor parenchyma. However, these mechanisms are often examined separately. How KRAS-driven cytokine changes couple with metabolic remodeling, and how metabolites reinforce stromal programs, remain unclear. More importantly, how these interactions converge on myeloid accumulation, CAF/ECM remodeling, and spatial T-cell exclusion remains insufficiently defined. Cytokine signaling and metabolic reprogramming are tightly interconnected in PDAC through shared transcriptional nodes and reciprocal feedback mechanisms, jointly sustaining immunosuppression and CD8^+^ T-cell exclusion. This review addresses how KRAS-driven signaling integrates cytokine and metabolic programs to shape immune exclusion in PDAC.

## 2 KRAS-driven cytokine networks

Oncogenic KRAS reshapes cytokine and chemokine production in PDAC cells, linking tumor-cell-intrinsic signaling to myeloid-cell recruitment, stromal remodeling, and immune suppression. KRAS-associated secretory programs include GM-CSF, CXCL family members, CCL2, IL-6, IL-33, and other inflammatory mediators, although individual cytokines differ in their direct KRAS dependence (Bayne et al., 2012; Pylayeva-Gupta et al., 2012; Alam et al., 2022; Li et al., 2022; McAndrews et al., 2022; Bianchi et al., 2023). These cytokine networks promote neutrophil, monocyte, and MDSC accumulation, activate CAF-related stromal programs, and reinforce STAT3- or NF-κB-associated inflammatory signaling within the TME (Feig et al., 2013; Li et al., 2022). Thus, KRAS-associated cytokine remodeling helps establish the immunosuppressive microenvironment and sets the stage for cytokine–metabolic crosstalk.

### 2.1 Secretome reprogramming

Oncogenic KRAS is a central driver of PDAC cell proliferation and reshapes the tumor-cell secretory program. Beyond sustaining tumor growth, oncogenic KRAS signaling reshapes the immune microenvironment. In pancreatic epithelial cells expressing oncogenic KRAS^G12D^, KRAS signaling induces GM-CSF, which promotes Gr-1^+^CD11b^+^ myeloid-cell accumulation and impairs CD8^+^ T-cell-mediated tumor control (Bayne et al., 2012; Pylayeva-Gupta et al., 2012). In parallel, KRAS-associated inflammatory circuits extend to CXCL1/2, CCL2, IL-6, and IL-1-related signaling, coordinating neutrophil and monocyte recruitment, MDSC expansion, CAF activation, and STAT3- or NF-κB-driven immunosuppressive remodeling in the PDAC TME (Biffi et al., 2019; Li et al., 2022; McAndrews et al., 2022; Bianchi et al., 2023). In addition, oncogenic KRAS^G12D^ enhances IL-33 expression in tumor cells, while intratumoral fungal signals further amplify IL-33 secretion and promote type 2 immune remodeling (Alam et al., 2022). IL-1 can activate CAFs and regulate their phenotypes through the JAK/STAT pathway (Biffi et al., 2019) and CXCL1 promotes neutrophil recruitment and sustains TNF-related inflammatory responses (Bianchi et al., 2023). Multi-kinase inhibition targeting this secretory network can enhance immune responses, further supporting its therapeutic plasticity (Falcomatà et al., 2022).

GM-CSF and CXCL chemokines are key components of the KRAS-related secretory network. GM-CSF promotes myeloid cell accumulation and induces the formation of immunosuppressive MDSCs (Takeuchi et al., 2015). CXCL1/2 and related chemokines contribute to neutrophil and myeloid cell recruitment and influence the spatial distribution of immune cells, causing them to remain preferentially in the tumor stroma rather than infiltrating the tumor parenchyma (Jeong et al., 2025; Bever et al., 2026). The IL-6–STAT3 axis further links inflammatory signaling with stromal remodeling, metabolic adaptation, and chemoresistance, and IL-6 receptor blockade can enhance chemotherapy efficacy in PDAC models (Wörmann et al., 2016; Long et al., 2017).

Clinical and single-cell studies also suggest that enhanced inflammatory signaling in PDAC is commonly associated with an immunosuppressive microenvironment and poor prognosis (Hosein et al., 2022). Complex interactions exist among tumor cells, CAFs, and immune cells, and specific CAF subsets may further contribute to immunosuppression and stromal remodeling (Dominguez et al., 2020; Chen M. M. et al., 2025). Overall, KRAS-associated secretory remodeling promotes myeloid recruitment through mediator-specific axes. Among these axes, the KRAS^G12D^–GM-CSF pathway provides direct evidence linking tumor-cell-intrinsic KRAS signaling to myeloid-cell accumulation and impaired T-cell control. Tumor-cell-derived GM-CSF induces Gr-1^+^CD11b^+^ myeloid-cell accumulation, reduces CD8^+^ T-cell infiltration, and supports pancreatic tumor development (Bayne et al., 2012; Pylayeva-Gupta et al., 2012). In KRAS-mutant pancreatic epithelial cells, PPARδ induces CCL2 secretion and recruits immunosuppressive macrophages and MDSCs through the CCL2/CCR2 axis (Liu et al., 2022). CXCL1-related signaling drives neutrophil-rich inflammation through a tumor–myeloid–stromal feed-forward circuit: tumor-cell-derived CXCL1 recruits neutrophils, whereas neutrophil-derived TNF amplifies CXCL1 expression in tumor cells and CAFs, reinforcing inflammatory CAF polarization and T-cell exclusion (Bianchi et al., 2023). IL-6 acts as a STAT3-linked stromal and inflammatory amplifier. IL-6-producing CAF subsets promote therapy resistance, whereas alpha-smooth muscle actin-positive (αSMA^+^) CAF-specific IL-6 deletion enhances gemcitabine efficacy and anti-PD-1 response in PDAC models (McAndrews et al., 2022). However, most mechanistic studies of KRAS-driven cytokine remodeling have been performed in KRAS^G12D^ models, and whether other KRAS alleles generate comparable secretory programs remains incompletely defined. These findings identify GM-CSF as a relatively direct tumor-cell-intrinsic KRAS-linked axis, whereas CCL2, CXCL1/2, and IL-6 are more strongly shaped by tumor–stromal–myeloid interactions and differ in cellular source, target-cell specificity, and direct KRAS dependence. This secretory network may also interact with glycolysis, lactate accumulation, and the acidic TME through nodes such as STAT3 and NF-κB, thereby laying the foundation for the cytokine–metabolic feedback loop and CD8^+^ T-cell exclusion.

### 2.2 Myeloid cell recruitment and polarization

KRAS-mediated secretome reprogramming promotes myeloid cell recruitment and drives their transition toward immunosuppressive phenotypes. These effects primarily involve cytokine-mediated recruitment processes rather than direct T-cell regulation. In PDAC, myeloid cells are broadly enriched and are commonly associated with limited effector T-cell infiltration and the formation of an immunosuppressive microenvironment (Hou et al., 2020). Among these factors, GM-CSF is a key driver of MDSC expansion, promoting local MDSC accumulation and suppressing T-cell function (Takeuchi et al., 2015). NF-κB-related signaling further enhances MDSC recruitment and immunosuppressive activity (Liu et al., 2023). In addition, CCL2, IL-6, and CSF family signaling mainly mediate monocyte recruitment and TAM differentiation, thereby promoting their transition toward immunosuppressive phenotypes (Sun et al., 2025). TAMs and MDSCs can also contribute to immunosuppression through TGF-β production, which promotes stromal activation, extracellular matrix remodeling, and T-cell exclusion in PDAC. In addition, IL-1β-producing myeloid cells, particularly TAMs, contribute to inflammatory remodeling and further reinforce immunosuppressive myeloid programs in the TME. These recruited myeloid cells further suppress T-cell immunity by depleting nutrients, producing reactive oxygen species and nitric oxide, and releasing immunosuppressive mediators.

Inflammatory signaling and metabolic regulation may cooperate during myeloid remodeling. For example, peroxisome proliferator-activated receptor δ (PPARδ) acts not only as a metabolic regulator but also as a promoter of myeloid cell infiltration and tumor progression through the CCL2 axis (Liu et al., 2022). This finding suggests that metabolic regulators can shape chemokine-dependent myeloid niches, rather than merely supporting tumor-cell metabolic fitness. At the neutrophil level, CXCL-related signaling is closely associated with increased neutrophil infiltration, immune exclusion features, and poor prognosis (Somani et al., 2026). Consistently, single-cell analysis identified BHLHE40-driven pro-tumor subsets of tumor-associated neutrophils (TANs) with hyperactivated glycolysis, suggesting that neutrophil recruitment and metabolic adaptation may jointly reinforce myeloid niches in the PDAC TME (Wang et al., 2023). Spatially, myeloid cells predominantly accumulate at tumor margins and stromal regions. This pattern is driven by chemokine networks and may also be influenced by the local metabolic environment. For example, lactate-rich conditions promote TAM recruitment and immunosuppressive signaling *via* STAT3/CCL2, while lactylation-driven N6-methyladenosine (m^6^A) modification may further enhance the suppressive activity of tumor-infiltrating myeloid cells (Xiong et al., 2022; Sun et al., 2025).

### 2.3 T-Cell suppression and the formation of immune exclusion

In the context of sustained myeloid cell enrichment, effector T cells in PDAC are not only reduced in number but also functionally suppressed. MDSCs and TAMs can inhibit CD8^+^ T-cell activity through arginine depletion, accumulation of reactive oxygen species (ROS) and nitric oxide, and the release of immunosuppressive mediators (Hezaveh et al., 2022; Somani et al., 2026). Blockade of CXCL1–CXCR2 signaling or reprogramming of TAMs can restore T-cell infiltration and enhance antitumor activity, further supporting the central role of myeloid cells in T-cell suppression (Bianchi et al., 2023; Rohila et al., 2023). In addition, MDSCs and TAMs can promote regulatory T cells (Tregs) expansion and maintain their immunosuppressive phenotype, while nutrient competition and metabolite accumulation further impair T-cell activation and differentiation (Hezaveh et al., 2022).

Immune exclusion also has a clear spatial basis. Histological and transcriptomic studies show that T cells are often confined to tumor margins or stromal regions, with limited access to the tumor core. In PDAC, T-cell exclusion is maintained by spatially organized stromal, chemokine, and metabolic constraints rather than by a single KRAS-driven barrier. At the stromal–chemokine level, fibroblast activation protein-positive (FAP^+^) CAF-derived CXCL12 coats tumor cells and blocks T-cell entry into cancer-cell regions; CXCL12–CXCR4 disruption restores T-cell access and sensitizes tumors to programmed death-ligand 1 (PD-L1) blockade (Feig et al., 2013). This finding supports a stromal mechanism of T-cell exclusion in KRAS-mutant PDAC. TGF-β-dependent LRRC15^+^ CAFs further define a stromal immunosuppressive program that suppresses CD8^+^ T-cell function and limits checkpoint blockade response, whereas LRRC15^+^ CAF depletion enhances CD8^+^ T-cell effector activity (Krishnamurty et al., 2022). A CXCL1–neutrophil–TNF loop further amplifies this exclusionary pattern by increasing CXCL1 expression in tumor cells and CAFs, supporting stromal inflammation and T-cell exclusion (Bianchi et al., 2023). Metabolic stress adds another layer of restriction. In PDAC cells, Solute carrier family 4 member 4 (SLC4A4) promotes extracellular acidosis and lactate accumulation; SLC4A4 inhibition reduces lactate production, increases CD8^+^ T-cell infiltration and interferon-γ (IFN-γ) production, and improves immune checkpoint blockade response (Cappellesso et al., 2022).

These findings indicate that CAF-derived chemokines, LRRC15^+^ myofibroblastic programs, CXCL1–neutrophil inflammation, and lactate/acidosis cooperate to restrict CD8^+^ T-cell infiltration and effector function, with variable dependence on tumor-intrinsic KRAS signaling (Feig et al., 2013; Cappellesso et al., 2022; Krishnamurty et al., 2022; Bianchi et al., 2023). CAF heterogeneity further shapes the stromal basis of immune exclusion. iCAFs are enriched in inflammatory cytokine and chemokine programs, including IL-6- and CXCL-related signaling, which may promote myeloid-cell recruitment and T-cell dysfunction (Biffi et al., 2019; McAndrews et al., 2022). MyCAFs are linked to extracellular matrix production, collagen remodeling, and stromal stiffness, forming a physical barrier that limits CD8^+^ T-cell infiltration into the tumor parenchyma (Chen et al., 2021; Krishnamurty et al., 2022; Liu et al., 2025c). In contrast, apCAFs display antigen-presentation-related features and may modulate local immune responses, although their role in PDAC immune exclusion remains context dependent (Elyada et al., 2019). Metabolic stress, including lactate accumulation, extracellular acidification, and hypoxia, may further reinforce CAF activation and ECM remodeling, linking metabolic reprogramming to cytokine-driven stromal exclusion (Cappellesso et al., 2022; Kitamura et al., 2023). Altered collagen architecture, CAF-related signaling, tumor-derived exosomes, and inflammatory signals can all reinforce this immune-excluded state (Quaranta et al., 2018; Chen et al., 2021).

### 2.4 Current controversies and research gaps

Although KRAS-driven cytokine networks have become an important framework for explaining immunosuppression in PDAC, the dominant signaling pathway remains unclear. Different studies have emphasized the roles of IL-6–STAT3 signaling (McAndrews et al., 2022), the CXCL chemokine axis (Bianchi et al., 2023), and NF-κB-related inflammatory pathways (Liu et al., 2023), suggesting that this network may be shaped by tumor stage, genetic background, and microenvironmental composition rather than by a single pathway. The cellular sources of these signals are also complex. In addition to tumor cells, CAFs, TAMs, and neutrophils can produce and amplify inflammatory signals. Distinct CAF subsets regulate immunosuppression and therapeutic responses through factors such as IL-6 (McAndrews et al., 2022), whereas myeloid cells can promote CXCL expression through NF-κB signaling and maintain the suppressive state (Liu et al., 2023). Moreover, EZH2-dependent secretome reprogramming can alter immune cell infiltration patterns, indicating that epigenetic regulation may also contribute to inflammatory secretory phenotypes (Chibaya et al., 2023).

Several controversies remain unresolved. First, the relative contribution of cytokine signaling and metabolic regulation to immune exclusion remains difficult to define. IL-6, CXCLs, and TNF-α promote suppressive myeloid recruitment and stromal inflammation, whereas lactate, extracellular acidity, and adenosine directly impair T-cell function and reinforce myeloid or stromal suppression. Second, CAFs have context-dependent roles. LRRC15^+^ or myCAF-like programs may restrict T-cell infiltration through ECM deposition, but depletion of selected fibroblast populations or collagen components can also aggravate immune suppression, indicating that CAFs are not uniformly tumor promoting (Dominguez et al., 2020; Chen et al., 2021; Krishnamurty et al., 2022). Third, inflammatory pathway hierarchy remains unclear, because IL-1/TGF-β, IL-6–STAT3, CXCL–CXCR2, and NF-κB-related circuits may dominate in different spatial niches, disease stages, or treatment states. Finally, whether KRAS-targeted therapy alone can reverse immune exclusion remains uncertain. KRAS^G12D^ inhibition shows that tumor-cell-intrinsic KRAS signaling can reshape the PDAC immune microenvironment, including CD8^+^ T-cell activity and myeloid-cell composition; however, advanced tumors may still require immune checkpoint blockade or additional TME-directed interventions (Mahadevan et al., 2023).

Additional examples further illustrate this complexity. CXCL1-driven neutrophil-derived TNF signaling can sustain inflammatory and immunosuppressive circuits (Bianchi et al., 2023), while ECM remodeling and CAF heterogeneity can regulate tumor progression and the immune microenvironment through molecules such as POSTN (Wu et al., 2025). These findings suggest that cytokine networks are closely coupled with stromal remodeling and metabolic states, but their temporal and spatial coordination with KRAS-related inflammatory networks remains unclear.

## 3 KRAS-driven metabolic networks

Oncogenic KRAS also reshapes metabolic programs in PDAC cells, linking tumor-cell-intrinsic signaling to nutrient scavenging, extracellular acidification, stromal metabolic exchange, and immune suppression. KRAS-associated metabolic programs include enhanced glycolytic flux, lactate accumulation, macropinocytosis, extracellular protein uptake, glutamine rewiring, stromal alanine support, and lipid mediator-associated inflammation (Commisso et al., 2013; Kamphorst et al., 2015). These metabolic networks can impair T-cell activity, reinforce myeloid and stromal suppression, and generate metabolite-driven inputs that interact with cytokine signaling within the TME (Colegio et al., 2014; Sousa et al., 2016).

### 3.1 Glucose metabolic reprogramming and lactate accumulation

KRAS-driven metabolic reprogramming in PDAC is characterized by enhanced glycolytic activity as a direct consequence of oncogenic KRAS signaling. KRAS mutations can upregulate glucose transporters such as glucose transporter 1 (GLUT1) and multiple glycolytic enzymes by regulating metabolic transcriptional programs, thereby promoting glucose uptake and aerobic glycolytic flux (Liu et al., 2021). Consistently, inducible KRAS^G12D^ extinction. Reduces glucose uptake and decreases glucose-intermediate flux into glycolysis, the hexosamine biosynthesis pathway, and the non-oxidative pentose phosphate pathway in pancreatic tumors. These findings indicate that oncogenic KRAS directly maintains anabolic glucose metabolism in native PDAC tumors (Ying et al., 2012). Under hypoxic conditions, KRAS can further support glycolysis through pathways such as hypoxia-inducible factor-1α (HIF-1α) and regulate pH homeostasis-related molecules, including carbonic anhydrase 9 (CA9), supporting tumor cell survival under metabolic stress (McDonald et al., 2019). Lactate links KRAS-driven glycolysis to microenvironmental reprogramming. Lactate accumulation is a major consequence of enhanced glycolysis and is associated with tumor progression and metastatic potential (Nimmakayala et al., 2021). This glycolysis–lactate axis links tumor-cell metabolism to inflammatory and immune remodeling, as lactate acts on myeloid and stromal cells as an immunoregulatory metabolite rather than merely serving as a metabolic end product.

Lactate also functions as an immunoregulatory signal and contributes to microenvironmental remodeling. It can modulate myeloid cell function through protein lactylation, promote TAM recruitment, and reinforce the immunosuppressive state (Sun et al., 2025). In PDAC, elevated protein lactylation promotes TAM recruitment and STAT3/CCL2-related immunosuppressive signaling, suggesting a link between metabolic and cytokine-dependent myeloid remodeling (Sun et al., 2025). Lactate may also enhance myeloid immunosuppressive activity through mechanisms such as m^6^A modification modification (Xiong et al., 2022). At the effector immune level, the acidic microenvironment can inhibit CD8^+^ T-cell activity and impair effector function, while lactate regulates macrophage metabolic states and polarization, promoting their transition toward immunosuppressive phenotypes (Cappellesso et al., 2022; Thielman et al., 2024). Lactate-rich and acidic conditions may promote CAF activation and ECM deposition, contributing to stromal remodeling associated with T-cell exclusion. Consistently, targeting lactate production or related metabolic pathways may improve the TME and enhance therapeutic responses (Cappellesso et al., 2022; Sun et al., 2025). Single-cell studies further show that enhanced glycolysis occurs not only in tumor cells but also in immune cells such as neutrophils, and is associated with pro-tumor phenotypes (Wang et al., 2023). Overall, KRAS-driven glucose metabolic reprogramming in PDAC cells increases glycolytic flux and lactate production, but its link to immune exclusion depends on defined immune-metabolic mechanisms rather than a direct KRAS-to-T-cell pathway. Tumor-derived lactate enhances MDSC immunosuppressive activity and contributes to treatment resistance, whereas glycolytic macrophages support PDAC progression through GLUT1-dependent NLRP3–IL-1β signaling; macrophage-specific GLUT1 deletion reduces tumor burden and increases CD8^+^ T-cell activity (Yang et al., 2020; Penny et al., 2021). Cancer-derived lactate is also reused by CAFs in glucose-poor PDAC regions, supporting stromal cell proliferation and sustaining a metabolically restricted TME (Kitamura et al., 2023).

### 3.2 Amino acid and lipid metabolic reprogramming

Beyond glucose metabolism, KRAS-mutant PDAC also remodels amino acid and lipid metabolism. Among these programs, the GOT1-dependent noncanonical glutamine pathway is directly linked to oncogenic KRAS signaling, whereas tryptophan metabolism, lipid mediator production, and nutrient-stress adaptations may reflect broader TME-driven metabolic remodeling. Glutamine metabolism is one of the most extensively studied pathways. In KRAS-mutant PDAC, tumor cells use a noncanonical glutamine pathway in which glutamine-derived aspartate is converted to oxaloacetate by GOT1, thereby maintaining redox balance (Son et al., 2013). Beyond enzyme-level glutamine rewiring, Ras-transformed pancreatic cancer cells acquire amino acids by macropinocytosing extracellular proteins, which are degraded to support growth under nutrient limitation (Commisso et al., 2013). However, not all nutrient inputs are tumor-cell-intrinsic KRAS outputs. Pancreatic stellate cells release alanine through autophagy, and cancer cells use this stromal alanine as a carbon source for the tricarboxylic acid cycle and biosynthetic metabolism in nutrient-poor PDAC niches (Sousa et al., 2016). SIRT5 deficiency can enhance this pathway by increasing GOT1 acetylation and promoting tumor progression (Hu et al., 2021). GOT2 also participates in metabolism and regulates PPARδ transcriptional activity, thereby influencing the tumor immune microenvironment (Abrego et al., 2022). In contrast, pathways involving tryptophan metabolism, ornithine/polyamine synthesis, or immune-cell amino acid metabolism may reflect broader TME-associated adaptation rather than direct KRAS regulation. In addition, ornithine aminotransferase (OAT)-mediated metabolism supports polyamine synthesis and tumor cell proliferation (Lee et al., 2023), while tryptophan metabolites can activate the aryl hydrocarbon receptor (AhR) in TAMs, linking amino acid metabolism to immune suppression (Hezaveh et al., 2022).

Lipid metabolism and related carbon sources also contribute to immune regulation. Acetate can serve as a carbon source for acetyl-CoA production and enhance c-Myc transcriptional activity by promoting c-Myc acetylation, thereby upregulating PD-L1, and glycolysis-related genes and promoting immune escape (Wang et al., 2024). Although the specific relevance of this mechanism to PDAC requires further validation, it suggests that lipid-related metabolism may connect metabolic adaptation with immune suppression. In PDAC, an inflammatory circuit can also form between tumor cells and IL-1β-secreting macrophages, and together with lipid mediators such as prostaglandin E2 (PGE2), this circuit contributes to a pathogenic inflammatory microenvironment (Caronni et al., 2023). Among amino acid- and lipid-related pathways, GOT1-dependent glutamine metabolism is a well-established KRAS-regulated program in PDAC cells that supports reduced nicotinamide adenine dinucleotide phosphate (NADPH) production, redox balance, and tumor-cell growth (Lyssiotis et al., 2013; Son et al., 2013). In contrast, tryptophan-derived AhR activation in TAMs, acetate-induced c-Myc–PD-L1 upregulation, and PGE2–IL-1β macrophage inflammation are better described as TME-associated metabolic–immune adaptations that reinforce immune suppression rather than uniformly KRAS-driven mechanisms (Hezaveh et al., 2022; Caronni et al., 2023; Wang et al., 2024).

### 3.3 Metabolic microenvironment and immunosuppression

In KRAS-mutant PDAC, metabolic remodeling involves both tumor-cell-intrinsic programs and TME-level adaptations. Enhanced glycolysis generates lactate and extracellular acidity, while stromal cells, myeloid cells, and pH-regulatory pathways shape metabolic niches that suppress immune activity. Lactate accumulation reflects a highly glycolytic state and may promote TAM recruitment and the formation of an immunosuppressive microenvironment through protein lactylation and STAT3/CCL2-related signaling (Sun et al., 2025). Meanwhile, lactate acts as an important mediator of metabolic crosstalk between tumor cells and CAFs by promoting H3K18 lactylation, CAF activation, and ECM deposition, thereby reducing CD8^+^ T-cell infiltration. This lactate–CAF axis links lactate signaling to ECM deposition and CD8^+^ T-cell exclusion *via* H3K18 lactylation (Ma Y. et al., 2026). In addition, acetate-related metabolic signaling can enhance c-Myc activity, upregulate PD-L1 and glycolysis-related genes, and promote immune evasion, suggesting that metabolic reprogramming can amplify immunosuppression through multiple routes (Wang et al., 2024). The acidic environment itself also contributes to immunosuppression. The regulation of extracellular pH is also shaped by tumor–microenvironment interactions rather than by tumor-cell-intrinsic KRAS signaling alone (McDonald et al., 2019). SLC4A4, helps maintain the acidic microenvironment of PDAC, and inhibition of this pathway reduces lactate production, alleviates local acidification, improves T-cell-mediated antitumor responses, and weakens macrophage-dominated immunosuppression (Cappellesso et al., 2022).

The metabolic microenvironment also cooperates with stromal remodeling to maintain immune exclusion. Specific CAF subsets, such as LRRC15^+^ CAFs, can form physical barriers by promoting fibrotic matrix deposition, thereby restricting CD8^+^ T-cell entry into the tumor core (Dominguez et al., 2020). LRRC15^+^ CAFs mark a TGF-β-activated myofibroblastic stromal state, indicating that immune exclusion is driven by specific CAF programs rather than a uniform CAF population (Dominguez et al., 2020; Krishnamurty et al., 2022). CAF-related signaling can also weaken antitumor immunity by regulating PD-L1 expression and immune checkpoint pathways (Koikawa et al., 2021), while stromal stiffness promotes PD-L1 membrane localization through the IGF2BP2–sphingolipid metabolism axis, reducing effector cell infiltration and enhancing immune escape (Tang et al., 2025). In addition, IL-17-induced neutrophil extracellular traps can restrict CD8^+^ T-cell infiltration and are associated with lactate alterations, linking inflammatory responses with local metabolic stress (Zhang et al., 2020).

### 3.4 Current controversies and pathway differences

Although KRAS-driven metabolic reprogramming is well established in PDAC, the pathways most relevant to immune exclusion remain unclear. Glycolysis, lactate accumulation, and extracellular acidification are linked to T-cell suppression and myeloid or stromal remodeling, whereas glutamine-dependent redox control, ornithine/polyamine synthesis, lipid metabolism, and lysosome-related adaptation may dominate in specific tumor states (Lee et al., 2023; Cheng et al., 2025). These pathways likely interact, and their relative importance may vary with nutrient availability, hypoxia, stromal density, and immune-cell composition. Glucose-poor or hypoxic regions may favor alternative nutrient use, whereas stroma-rich regions may support metabolic exchange between stromal and tumor cells. Thus, distinct metabolic programs may operate in different spatial regions of PDAC.

Furthermore, emerging evidence of allele-specific metabolic rewiring suggests that KRAS-driven metabolism should not be viewed as a uniform process. Different KRAS variants may also be associated with distinct glucose and glutamine metabolic profiles as well as different immune states, indicating marked molecular heterogeneity and context dependence in the metabolic network of PDAC (Ardalan et al., 2025). For example, KRAS^G12R^ differs from KRAS^G12D^ and KRAS^G12V^ in PI3K pathway engagement and macropinocytosis, which may create allele-specific metabolic dependencies (Hobbs et al., 2020). Therefore, KRAS^G12D^, KRAS^G12V,^ and KRAS^G12R^ should not be assumed to drive identical metabolic programs or immune phenotypes. The mechanisms by which KRAS regulates metabolism are also diverse. KRAS can control metabolic gene expression through transcriptional programs such as MYC (Zhang et al., 2024), while key metabolic nodes may directly alter metabolic flux distribution and influence therapeutic responses. For example, the SREBP1–PCSK9 axis regulates immune escape through lipid metabolic reprogramming and lysosomal degradation of PD-L1, thereby affecting responses to immunotherapy (Lao et al., 2025).

Whether targeting metabolic vulnerabilities alone is sufficient to reverse the immune-excluded phenotype of PDAC remains unresolved. The effects of metabolic alterations on immunity are not uniformly suppressive. On the one hand, autophagy and metabolic adaptation can weaken antigen presentation and enhance immune escape by promoting major histocompatibility complex class I (MHC-I) degradation (Yamamoto et al., 2020). On the other hand, certain therapy-induced state transitions can improve vascular perfusion and promote CD8^+^ T-cell infiltration, thereby enhancing responses to immunotherapy (Ruscetti et al., 2020). Specific inflammatory–metabolic environments are also associated with tertiary lymphoid structure formation and better prognosis (Amisaki et al., 2025), suggesting context-dependent bidirectional regulation between metabolism and immunity. Functional perturbation will still be needed to distinguish driver nodes from associated changes. Glutamine antagonism targets PDAC metabolic dependencies and modulates suppressive myeloid cells, whereas SLC4A4 inhibition reduces lactate production and acidosis and improves T-cell-mediated responses (Cappellesso et al., 2022; Encarnación-Rosado et al., 2024). However, metabolic interventions often affect tumor and immune cells simultaneously, and compensatory nutrient acquisition, such as nuclear factor erythroid 2-related factor 2 (NRF2)-dependent macropinocytosis after autophagy inhibition, may limit single-agent efficacy (Su et al., 2021).

Representative cytokine–metabolic pathways involved in PDAC immune exclusion are summarized in Table 1.

**TABLE 1:** KRAS-associated cytokine–metabolic pathways in PDAC immune exclusion.

Core axis/Pathway	Main cell types	Upstream input	Directional mechanism	Immune consequence	Evidence level
KRAS–GM-CSF axis	Tumor cells, myeloid cells	Tumor cell-intrinsic KRAS^G12D^ signaling	Oncogenic KRAS directly induces GM-CSF secretion → drives expansion and activation of Gr-1^+^CD11b^+^ myeloid-derived suppressor cells	Accumulation of suppressive myeloid cells; restricted CD8^+^ T-cell-mediated tumor control	High: Direct genotype-linked evidence
KRAS–NF-κB–CXCLs axis	Tumor cells, MDSCs, neutrophils	Oncogenic KRAS *via* CRIP1 or IRAK2 signaling	KRAS activation facilitates NF-κB (p65) nuclear translocation → transcriptionally upregulates CXCL1, CXCL2, and CXCL5 → recruits CXCR2^+^ myeloid populations	Local enrichment of MDSCs and neutrophils; blocks spatial infiltration of effector CD8^+^ T cells	Moderate: Functional TME evidence
Lactate–STAT3/CCL2–TAM axis	Tumor cells, TAMs	KRAS-driven glycolytic rewiring (HIF-1α/MYC)	Accelerated glycolysis leads to extracellular lactate accumulation and acidosis → activates STAT3/CCL2 cascade and feedback loop (including ENSA-mediated pathways)	Drives suppressive M2-like macrophage polarization and recruitment; dampens T-cell proliferation and IFN-γ production	Moderate: Functional TME evidence
Lactate–H3K18la–CAF/ECM axis	CAFs, tumor cells	Tumor-derived lactate secretion	Extracellular lactate uptake by CAFs triggers histone H3K18 lactylation (H3K18la) → epigenetically activates pro-fibrotic gene programs and collagen deposition	Promotes dense ECM remodeling remodeling; establishes a physical barrier that spatially excludes CD8^+^ T cells	Moderate: Functional TME evidence
CD39/CD73–Adenosine axis	Tumor cells, Tregs, CD8^+^ T cells	Microenvironmental hypoxia and metabolic stress	Hypoxic stress coupled with extracellular ATP degradation activates CD39 and CD73 → triggers profound adenosine accumulation → engages A2A/A2B receptors on T cells	Suppresses CD8^+^ T-cell activation, cytolytic activity, and IFN-γ secretion	Moderate: Functional TME evidence
Tryptophan–AhR–Myeloid axis	TAMs, tumor cells	Altered amino acid catabolism in the TME	Accumulation of tryptophan-derived catabolites activates the aryl hydrocarbon receptor (AhR) in myeloid lineages	Promotes local immunosuppressive macrophage states and weakens antitumor T-cell activity	Moderate: Functional TME evidence

Evidence levels indicate the strength of KRAS-related and immune functional evidence.

## 4 Cytokine–metabolic cooperative networks and the formation of the immune-excluded phenotype

In KRAS-mutant PDAC, cytokine and metabolic programs are functionally interconnected and collectively shape tumor–microenvironment interactions. Tumor-cell-intrinsic KRAS signaling contributes to inflammatory and metabolic outputs, including cytokine secretion, enhanced glycolytic activity, lactate production, and nutrient scavenging (Bayne et al., 2012; Pylayeva-Gupta et al., 2012; Commisso et al., 2013; Kamphorst et al., 2015). These tumor-derived inputs interact with multiple TME-associated processes, including myeloid-cell recruitment, CAF activation, hypoxia, extracellular acidification, adenosine signaling, and lipid mediator-associated inflammation (Feig et al., 2013; Colegio et al., 2014; Sousa et al., 2016; Graziano et al., 2023).

### 4.1 KRAS links cytokine signaling and metabolic reprogramming

**FIGURE 1:**
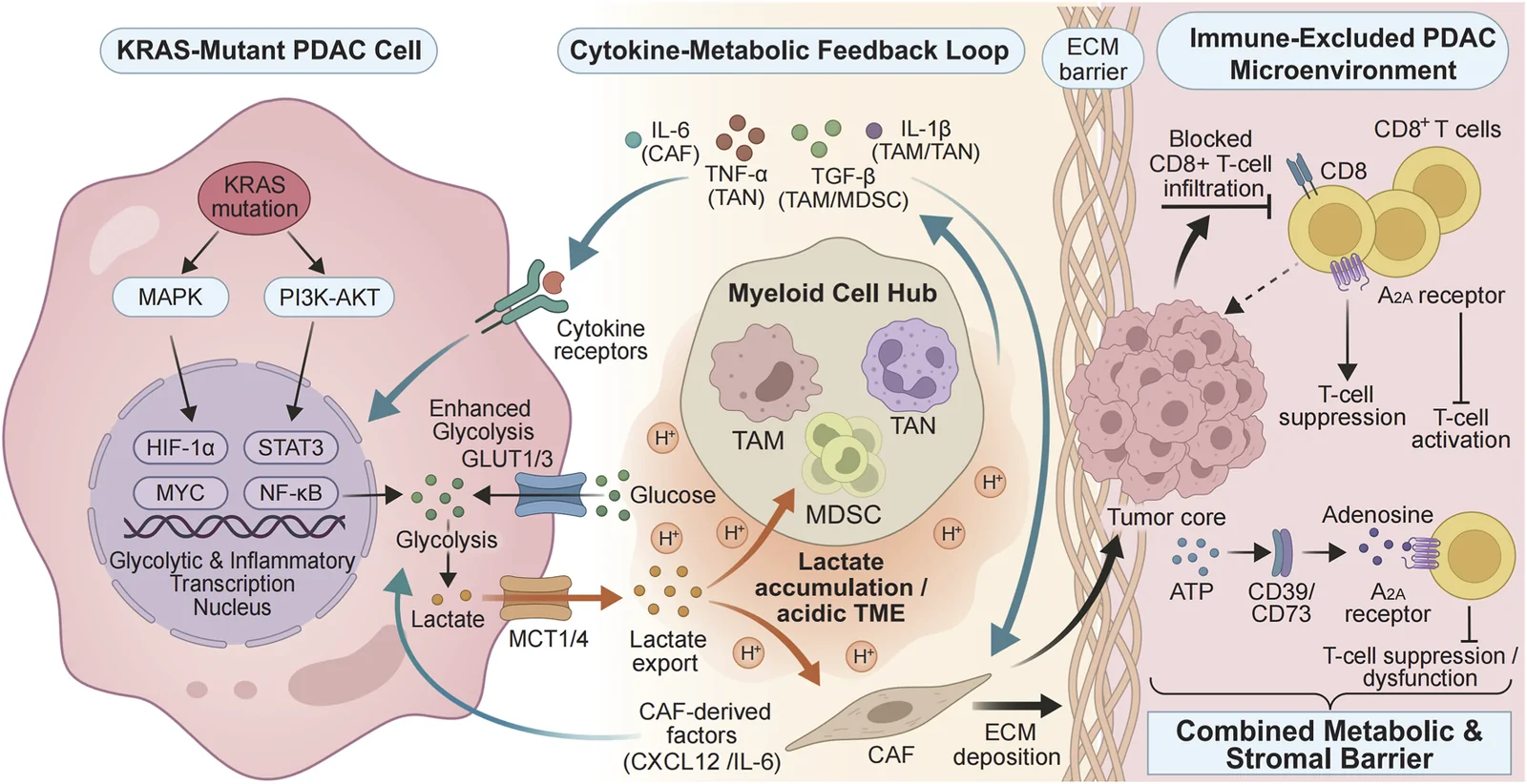
Cytokine–metabolic feedback loop underlying immune exclusion in KRAS-mutant PDAC. KRAS mutation activates MAPK and PI3K–AKT signaling in PDAC cells and promotes downstream transcriptional programs involving HIF-1α, STAT3, MYC, and NF-κB. These signals enhance glycolysis, increase GLUT1/3-mediated glucose uptake, and promote lactate export through MCT1/4. In parallel, KRAS-associated cytokine networks involve tumor-cell-derived mediators and microenvironment-derived cytokines. Tumor-cell-associated mediators, including GM-CSF and CXCLs, together with stromal- and immune-cell-derived cytokines, including IL-6 (CAF), TNF-α (TAN), TGF-β (TAM/MDSC), and IL-1β (TAM/TAN), further reinforce glycolytic and inflammatory programs. Lactate accumulation, extracellular acidification, and inflammatory cytokine signaling together support immunosuppressive myeloid-cell accumulation, CAF activation, and ECM deposition. This cytokine–metabolic remodeling promotes a combined metabolic and stromal barrier that restricts CD8^+^ T-cell entry into the tumor core. ATP-derived adenosine generated through CD39/CD73 further suppresses CD8^+^ T-cell activation and function via A2A receptor signaling. Together, cytokine signaling, metabolic remodeling, myeloid cell accumulation, and ECM deposition establish a combined metabolic and stromal barrier, leading to an immune-excluded PDAC microenvironment. PDAC, pancreatic ductal adenocarcinoma; TAM, tumor-associated macrophage; TAN, tumor-associated neutrophil; MDSC, myeloid-derived suppressor cell; CAF, cancer-associated fibroblast; ECM, extracellular matrix; TME, tumor microenvironment; GLUT, glucose transporter; MCT, monocarboxylate transporter; ATP, adenosine triphosphate; A2A receptor, adenosine A2A receptor.

Cytokine–metabolic crosstalk in PDAC reflects bidirectional regulation between inflammatory signaling and metabolic remodeling. KRAS functions as the upstream driver that integrates these inflammatory and metabolic programs in PDAC. Cytokine and chemokine signaling activates STAT3-, NF-κB-, and HIF-1α-dependent programs coupling inflammation with glycolytic and myeloid metabolic adaptation (Dey et al., 2020). In turn, lactate, adenosine, and lipid mediators reinforce myeloid recruitment, CAF/ECM remodeling, and CD8^+^ T-cell dysfunction (Cappellesso et al., 2022; Graziano et al., 2023; Sun et al., 2025). In PDAC, KRAS not only regulates inflammatory signaling and metabolic reprogramming separately but also connects these processes through shared signaling nodes (Figure 1 **)**. Upon activation, KRAS engages MAPK, PI3K–AKT–mTOR, NF-κB, and STAT3 pathways, while modulating transcriptional programs involving MYC, HIF-1α, and activating transcription factor 4 (ATF4). Through these nodes, tumor cell secretory programs, glycolysis, amino acid utilization, lipid metabolism, and immune regulation become interconnected.

HIF-1α is an important node linking hypoxic adaptation, enhanced glycolysis, and maintenance of the acidic microenvironment. Under hypoxic conditions, KRAS can promote HIF-1α/hypoxia-inducible factor-2α (HIF-2α) stabilization and upregulate CA9, MCT4, and GLUT1, thereby coordinating glycolysis, lactate export, and pH homeostasis. High CA9 expression is associated with poor prognosis and therapeutic resistance in PDAC, and its inhibition can improve therapeutic responses (McDonald et al., 2019).

MYC can also connect cytokine signaling with metabolic reprogramming. KRAS-mutant tumor cells can express the IL-2 receptor γ chain, IL-4Rα, and IL-13Rα1 complex, enabling them to respond to type II cytokines from Th2 cells. This Janus kinase 1 (JAK1)–STAT6-dependent signaling upregulates MYC and promotes glycolytic reprogramming (Dey et al., 2020). Therefore, MYC may function not only as a metabolic regulator but also as a bridge linking cytokine responses, inflammatory factor expression, and myeloid cell recruitment.

STAT3 further integrates inflammatory signaling with adaptive signaling responses in PDAC. It can be activated by cytokine inputs such as IL-6 and may also serve as a compensatory pathway after RAS–MEK pathway inhibition. In PDAC models, MEK inhibition induced rapid STAT3 activation, whereas combined MEK and STAT3 inhibition suppressed downstream signaling, reduced tumor growth and improved survival, suggesting that STAT3-mediated bypass activation contributes to resistance to RAS pathway inhibition (Nagathihalli et al., 2018). Overall, tumor-cell-intrinsic KRAS signaling may intersect with TME-derived cytokine inputs centered on HIF-1α, MYC, STAT3, and NF-κB, linking cytokine signaling with metabolic reprogramming and providing a mechanistic basis for downstream myeloid enrichment, stromal remodeling, and immune exclusion.

### 4.2 Cytokine signaling drives metabolic reprogramming

Cytokine signaling reshapes metabolic programs in both tumor and immune cells in PDAC. Inflammatory mediators activate STAT3-, NF-κB-, and HIF-1α-related pathways, increasing glucose uptake, glycolysis, lactate production, and myeloid-cell metabolic adaptation. The interleukin-8 (IL-8)–STAT3 axis provides a representative example. IL-8 secreted by TAMs promotes STAT3 phosphorylation and nuclear translocation, leading to increased GLUT3 expression, enhanced glucose uptake, and elevated lactate production in tumor cells. Blockade of the IL-8 receptor can inhibit this process and delay tumor progression (Zhong et al., 2024).

STAT3 can also integrate epigenetic and metabolism-related signals. VIRMA stabilizes STRA6 mRNA through m^6^A modification, thereby activating the STRA6–STAT3–HIF-1α axis, upregulating key glycolytic enzymes, and promoting tumor progression (Yang et al., 2024). Lactate-induced ENSA-K63 lactylation similarly enhances TAM recruitment and immunosuppression through STAT3/CCL2 signaling (Sun et al., 2025). In addition to STAT3, NF-κB is involved in metabolic regulation. IRAK2 is highly expressed in PDAC and maintains the glycolytic phenotype and tumor cell proliferation by activating NF-κB; inhibition of NF-κB can reverse these metabolic abnormalities and growth advantages (Yang et al., 2022).

At the immune cell level, cytokines also regulate myeloid cell proliferation and metabolic states. CAF-derived CSF1 induces high p21 expression in TAMs, promoting TAM proliferation and conferring an immunosuppressive phenotype. Chemotherapy can further enhance this effect and aggravate immunosuppression (Zuo et al., 2023). In addition, CTCF activates IGF2BP2 through an FLG-AS1-dependent mechanism and enhances CSF1 mRNA stability, thereby promoting the transition of TAMs toward an immunosuppressive phenotype (Liu et al., 2025b). Cytokine signaling coordinates metabolic reprogramming across tumor and myeloid compartments.

### 4.3 Metabolites reciprocally amplify immunosuppressive signaling

Metabolic reprogramming generates immunoregulatory metabolites that reinforce immune and stromal suppression in PDAC. Lactate, adenosine, and lipid mediators act on myeloid cells, CAFs, and T cells, amplifying immunosuppressive signaling and sustaining immune exclusion. Among these metabolites, lactate directly links tumor-cell-intrinsic KRAS signaling to metabolite-driven immune suppression. Oncogenic KRAS maintains glucose uptake and glycolytic/anabolic glucose flux in pancreatic tumors, supporting tumor-cell-derived lactate production and extracellular lactate accumulation (Ying et al., 2012). Lactate is one of the most important mediators in this process. Enhanced glycolysis in PDAC promotes lactate export through MCT1/MCT4, leading to local lactate accumulation and acidification. A high-lactate environment can suppress CD8^+^ T-cell proliferation, cytotoxicity, and IFN-γ secretion, while supporting Treg maintenance and inducing macrophage polarization toward an immunosuppressive phenotype (Cappellesso et al., 2022; Zhong et al., 2024). At the molecular level, lactate can further enhance immunosuppressive signaling through lactylation. After entering tumor-associated schwann cells (TASCs), lactate induces METTL16 lactylation and enhances m^6^A-dependent CTCF stability, thereby upregulating immunosuppression-related ligand expression and impairing CD8^+^ T-cell function (Liu et al., 2025a). Lactate-induced ENSA-K63 lactylation in tumor cells can also promote TAM recruitment and reinforce immunosuppression through STAT3/CCL2 signaling (Sun et al., 2025).

Adenosine and lipid mediators also amplify immunosuppression, but their links to tumor-cell-intrinsic KRAS metabolism appear less direct than lactate. CD39/CD73-mediated adenosine signaling represents a TME-level immunosuppressive pathway in PDAC. Enhanced ATP degradation sustains local adenosine accumulation, which suppresses T-cell activation, proliferation, and cytokine secretion through adenosine receptor signaling (Graziano et al., 2023). CD73-targeted inhibition can enhance CD8^+^ T-cell infiltration and prolong survival, whereas tumor-derived CXCL5 can induce MDSC chemotaxis through an AMP-dependent mechanism and partially offset this therapeutic effect; combined CXCR2 inhibition can further enhance antitumor activity (Chen et al., 2023). Lipid-related signaling also regulates immune escape. Stromal stiffness can stabilize IGF2BP2 and upregulate SGMS2, promoting PD-L1 localization in membrane lipid rafts and enhancing immune evasion (Tang et al., 2025). PGE2 also reflects inflammatory TME remodeling rather than a direct KRAS-dependent metabolic output. PGE2 inhibits T-cell effector activity through EP2/EP4 signaling; in pancreatic cancer models, this axis reduces mesoCAR T-cell proliferation, cytotoxicity, and IFN-γ/IL-2 production, whereas EP2/EP4 blockade restores these antitumor functions (Akbari et al., 2023). Meanwhile, PGE2 cooperates with TNF to induce IL-1β^+^ TAM subsets, forming an inflammatory circuit that promotes tumor progression (Caronni et al., 2023).

Metabolism-related changes can further influence the immune environment through epigenetic regulation and cell-state remodeling. Single-cell studies have identified a terminally differentiated pro-tumor TAN subset characterized by markedly enhanced glycolytic activity and driven by BHLHE40 (Wang et al., 2023). CLDN18 promotes ALCAM recruitment to membrane lipid rafts, enhancing immune synapse formation between tumor cells and cytotoxic T lymphocytes (CTLs) and thereby affecting T-cell infiltration (De Sanctis et al., 2024). Overall, lactate, adenosine, and lipid metabolites can convert metabolic alterations into immunosuppressive signals through receptor signaling, post-translational modifications, epigenetic regulation, and membrane remodeling, thereby sustaining the immunosuppressive microenvironment across multiple cell types.

### 4.4 From cooperative interactions to the formation of the immune-excluded phenotype

Several linked cytokine–metabolic pathways reflect this cooperative pattern in PDAC. TAM-derived IL-8 activates STAT3 signaling in tumor cells, increases GLUT3 expression, and enhances glucose uptake and lactate production (Zhong et al., 2024). In turn, lactate accumulation promotes TAM recruitment and immunosuppressive polarization through lactylation-related mechanisms and STAT3/CCL2 signaling (Sun et al., 2025). CD39/CD73-dependent adenosine suppresses CD8^+^ T-cell activation through A2A receptor signaling (Graziano et al., 2023), whereas acidic and lactate-rich conditions promote CAF activation and ECM deposition (Cappellesso et al., 2022; Ma Y. et al., 2026). These pathways promote myeloid enrichment and stromal restriction, limiting CD8^+^ T-cell infiltration. PDAC is usually not characterized by a complete absence of immune cells, but by myeloid cell enrichment, restricted effector T-cell infiltration, impaired antigen presentation, and spatial immune exclusion. In this process, immunosuppressive signals and structural barriers overlap, jointly limiting CD8^+^ T-cell entry into the tumor parenchyma and promoting the formation of an immune-excluded microenvironment.

Myeloid cell enrichment is a major effector component of immune exclusion. CRIP1 is highly expressed in PDAC and upregulates CXCL1/5 by enhancing NF-κB/p65 nuclear translocation, thereby driving MDSC recruitment. Inhibition of CXCR1/2 reduces MDSC infiltration, improves CD8^+^ T-cell entry into tumor tissues, and enhances responses to immunotherapy (Liu et al., 2023). CXCL1-mediated recruitment of neutrophil-like MDSCs further restricts the spatial distribution of T cells, while neutrophil-derived TNF amplifies CXCL1 expression through TNFR2 and promotes inflammatory polarization of CAFs, leading to continuous reinforcement of inflammatory signaling and immunosuppression (Bianchi et al., 2023). Mutant p53 can also enhance CXCL1 transcriptional activity and cooperate with NF-κB signaling to maintain an immunosuppressive microenvironment and weaken the efficacy of immune checkpoint blockade (Mahat et al., 2025).

Spatial immune exclusion is further maintained by multiple structural and molecular barriers. Cross-linked fibrin in the stroma can form a physical barrier that restricts CD8^+^ T cells and myeloid cells from entering the tumor parenchyma, whereas interfering with fibrin formation improves immune cell infiltration and delays tumor progression (Karim et al., 2025). TAM-derived extracellular vesicles promote H3K79 methylation-dependent ATG5 transcription through the CAV1–DOT1L axis, driving vasculogenic mimicry and aggravating immune exclusion (Liu et al., 2026). Tumor-intrinsic features also participate in this process. For example, CLDN18 regulates immune synapse formation by controlling ALCAM localization in membrane lipid rafts, thereby modulating T-cell infiltration (De Sanctis et al., 2024). In addition, perineural invasion, a common feature of PDAC, is closely linked to immunosuppression, involving CAFs, TAMs, and signaling pathways related to CXCL12/CXCR4, TGF-β, and neurotrophic factors (Xu et al., 2025). Overall, immune exclusion is not driven by a single molecule or pathway, but gradually emerges from the combined effects of cytokine signaling, metabolic reprogramming, stromal barriers, and nerve–immune–stromal interactions.

### 4.5 Maintenance mechanisms, heterogeneity, and therapeutic implications of immune exclusion

Sustained cytokine–metabolic feedback may maintain immune exclusion in PDAC. KRAS-mutant tumor cells produce GM-CSF and selected chemokines, whereas stromal and immune cells provide IL-6, TNF-α, TGF-β, and CXCL-related inflammatory inputs. These signals recruit myeloid cells and sustain STAT3-, NF-κB-, and HIF-related programs. Meanwhile, lactate, adenosine, and lipid metabolites favor immunosuppressive cell states and limit effector T-cell recovery. These feedback loops may help maintain the immune-excluded phenotype.

Cellular and metabolic heterogeneity further reinforces this stability. Single-cell transcriptomic analysis has identified a malignant epithelial subset enriched in advanced-stage and metastatic lesions, characterized by an immune-cold phenotype, reduced immune cell communication, increased Tregs, and effector T-cell exclusion. This subset shows enriched RARRES1 expression and is associated with chemotherapy resistance and increased KRAS mutation frequency (Ge et al., 2026).

Organoid-based metabolic classification has further divided PDAC into high-glucose-metabolism and high-lipid-metabolism subtypes. The former is associated with chemotherapy resistance and poor prognosis, with the GLUT1/ALDOB/G6PD axis contributing to this resistant phenotype (Li et al., 2023). Lipid metabolism-related molecules such as FASN also display tumor type- and stage-dependent differences (Chianese et al., 2023), while IL-6R/STAT3 signaling can dynamically switch at the single-cell level and drive metabolic phenotype changes (Blaszczak et al., 2024). TANs also show marked heterogeneity; the BHLHE40-driven TAN-1 subset exhibits high glycolytic activity and is associated with poor prognosis (Wang et al., 2023). These findings indicate that the immunometabolic network is not static but dynamically remodeled according to cellular states, metabolic dependencies, and therapeutic pressure.

These features may partly explain the limited efficacy of single-agent immune checkpoint blockade in PDAC. Multi-node combination strategies may be more suitable for disrupting stable immune exclusion. For example, simultaneous targeting of 4-1BB, LAG3, and CXCR1/2 enhances T-cell activation while relieving myeloid cell-mediated immunosuppression, leading to durable remission in PDAC models (Gulhati et al., 2023). CD73-mediated adenosine signaling contributes to immunosuppression in basal-like/squamous PDAC, suggesting the value of subtype-guided targeting (Faraoni et al., 2023). In addition, the CCL18/NF-κB/VCAM-1 axis forms a positive feedback loop between tumor cells and TAMs and drives metabolic reprogramming (Ye et al., 2018), while multi-target tyrosine kinase inhibitors combined with chemotherapy and immunotherapy have also shown therapeutic activity (Yang et al., 2025). KRAS-related tumor-cell programs and TME-derived cytokine–metabolic feedback may maintain immune exclusion through cellular heterogeneity and treatment-driven remodeling. Therapeutic strategies may therefore need to target selected regulatory modules according to patient-specific microenvironmental states.

## 5 Therapeutic implications

### 5.1 Limitations of single-agent therapeutic strategies

Although immunotherapy has made substantial progress in various solid tumors, its efficacy in PDAC remains limited. Immune checkpoint inhibitors used as monotherapy usually fail to significantly improve prognosis, mainly because PDAC lacks sufficient immune activation and typically exhibits an immune-excluded phenotype. Insufficient effector T-cell infiltration, enrichment of myeloid suppressive cells, and fibrotic stromal barriers jointly restrict antitumor immune responses. Even when tumor-reactive T cells are present, they often have difficulty penetrating the stroma and establishing effective contact with tumor cells (Li et al., 2021).

**FIGURE 2:**
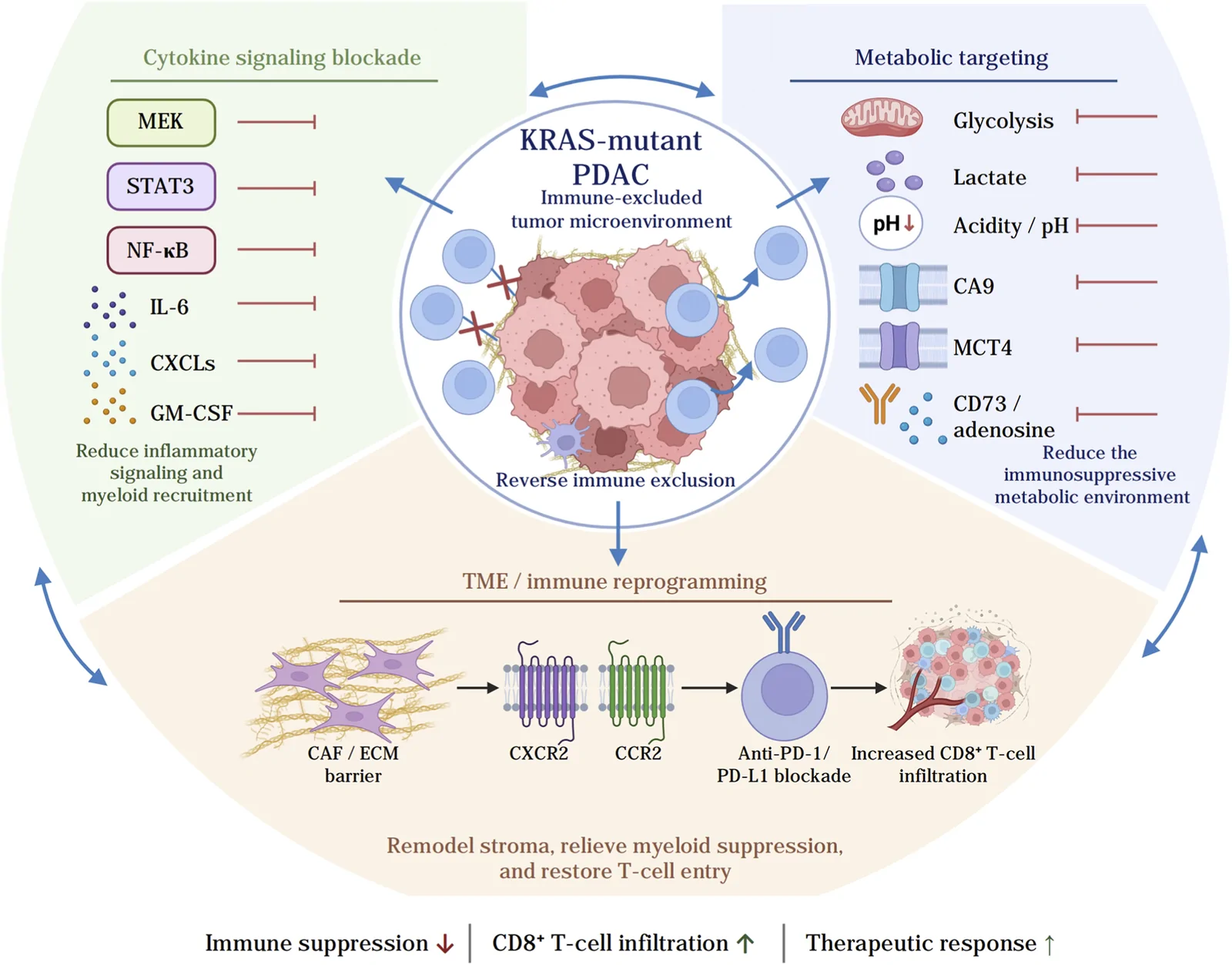
Combination strategies to reverse KRAS-driven immune exclusion in PDAC. KRAS-mutant PDAC maintains an immune-excluded tumor microenvironment through inflammatory signaling, metabolic suppression, stromal barriers, and myeloid cell recruitment. Targeting cytokine signaling nodes, including MEK, STAT3, NF-κB, IL-6, CXCLs, and GM-CSF, may reduce inflammatory signaling and myeloid recruitment. Metabolic targeting of glycolysis, lactate accumulation/export, extracellular acidity, CA9, MCT4, and the CD73/adenosine axis may reduce the immunosuppressive metabolic environment. In parallel, TME and immune reprogramming strategies, including weakening CAF/ECM-associated barriers, inhibiting CXCR2-and CCR2-mediated myeloid trafficking, and combining these approaches with anti-PD-1/PD-L1 blockade, may restore CD8^+^ T-cell entry and improve antitumor responses. PDAC, pancreatic ductal adenocarcinoma; CAF, cancer-associated fibroblast; ECM, extracellular matrix; CA9, carbonic anhydrase 9; MCT, monocarboxylate transporter; PD-1, programmed cell death protein 1; PD-L1, programmed death-ligand 1.

Single metabolic or inflammatory targeting strategies are also unlikely to achieve durable efficacy because compensatory mechanisms and positive feedback loops are common in the PDAC TME. Focal adhesion kinase (FAK) inhibition can reduce fibrosis and increase CD8^+^ T-cell infiltration, but its survival benefit as monotherapy is limited and usually requires combination with immune checkpoint inhibitors to enhance therapeutic efficacy (Jiang et al., 2016). IL-1β targeting shows differential responses across metastatic models, suggesting that its effects depend on organ-specific microenvironments. Inhibition of CCR2 or CXCR2 alone may also lead to compensatory recruitment of other myeloid cell subsets, thereby maintaining the immunosuppressive state (Nywening et al., 2018). Similarly, targeting the SREBP1–PCSK9 axis can enhance antitumor activity when combined with PD-1 blockade (Lao et al., 2025), while CDA- or P2Y6-related interventions require combination with anti-PD-1 therapy to generate stronger synergistic effects (Scolaro et al., 2024). In addition, after autophagy inhibition, PDAC cells can maintain survival by acquiring extracellular nutrients through NRF2-driven macropinocytosis, illustrating another compensatory route that limits single-agent efficacy (Su et al., 2021). These findings indicate that PDAC immunosuppression is not maintained by a single pathway. Instead, KRAS-related cytokine signaling and metabolic reprogramming interact to stabilize immune exclusion. Therefore, single-agent therapies are unlikely to simultaneously relieve myeloid suppression, metabolic barriers, and stromal restriction, supporting the need for multi-target combination strategies. Figure 2 summarizes a multi-layered therapeutic framework targeting inflammatory signaling, metabolic suppression, stromal barriers, myeloid recruitment, and immune checkpoints.

### 5.2 Combination intervention strategies based on the cooperative network

Given the close interaction between cytokine signaling and metabolic reprogramming in PDAC, single-pathway intervention is unlikely to produce durable therapeutic efficacy. The core rationale of combination therapy is to simultaneously target multiple mutually supportive regulatory nodes and disrupt the immunosuppressive homeostasis of the TME. KRAS/RAS-directed therapy may provide a genotype-defined and clinically actionable entry point for translational combinations in PDAC. In preclinical KRAS^G12D^-mutant PDAC models, the selective KRAS^G12D^ inhibitor MRTX1133 suppresses tumor-cell-intrinsic oncogenic signaling and remodels the TME by increasing intratumoral CD8^+^ effector T cells, reducing suppressive myeloid infiltration, reprogramming cancer-associated fibroblasts, and enhancing immune checkpoint blockade response (Mahadevan et al., 2023). These findings support KRAS blockade as a therapeutic test of the cytokine–metabolic crosstalk concept, suggesting that tumor-cell KRAS inhibition may weaken inflammatory and metabolic inputs that sustain myeloid recruitment, stromal activation, and T-cell dysfunction. Nevertheless, these observations are primarily derived from KRAS^G12D^ models, and whether other KRAS alleles exhibit comparable microenvironmental remodeling or therapeutic responses to allele-specific inhibition requires further investigation. RAS in the active GTP-bound state (RAS^ON)^ multi-selective inhibition with RMC-6236 suppresses active RAS signaling and shows antitumor activity in RAS-addicted models, including KRAS-mutant PDAC (Holderfield et al., 2024; Jiang et al., 2024). Clinically, daraxonrasib (RMC-6236) showed activity in previously treated RAS-mutated PDAC in a phase 1/2 study and improved overall and progression-free survival *versus* chemotherapy in the phase 3 RASolute 302 trial (O’Reilly et al., 2026; Wolpin et al., 2026). Therefore, KRAS/RAS inhibition may serve as a rational backbone for biomarker-guided combinations, with metabolic, stromal, or myeloid-targeting strategies selected according to dominant TME features, such as lactate accumulation, acidic pH, adenosine-high states, CAF-ECM barriers, or myeloid enrichment. Combined blockade of inflammatory signaling is one important direction. MEK and STAT3 pathways cooperate to maintain immunosuppressive stroma and cytokine networks in PDAC, and their combined inhibition can reshape CAF states, enhance T-cell infiltration, and improve responses to immunotherapy (Datta et al., 2022). However, MEK–STAT3 co-inhibition may not be suitable as an unselected combination strategy, because both pathways can affect T-cell function and stromal homeostasis. Its translational value may therefore depend on identifying tumors with high MAPK–STAT3 activity, inflammatory CAF programs, and poor CD8^+^ T-cell infiltration. Combination strategies integrating metabolic regulation with immunotherapy are also highly relevant. Enhanced glycolysis in tumor cells leads to lactate accumulation, which promotes CAF activation and stromal deposition through lactylation and restricts CD8^+^ T-cell infiltration (Sun et al., 2025; Ma Y. et al., 2026). Targeting pH-regulatory transporters such as SLC4A4 can improve the acidic microenvironment, restore T-cell function, and enhance the efficacy of immune checkpoint inhibitors (Cappellesso et al., 2022). Adenosine-axis blockade represents another actionable metabolic–immune strategy. CD73-dependent adenosine signaling through ADORA2B drives immunosuppression in ductal pancreatic cancer, suggesting that CD73-targeted approaches may be particularly relevant in adenosine-high tumors or basal-like/squamous immune-suppressed states (Faraoni et al., 2023). This strategy may be best positioned in biomarker-selected combinations, especially when adenosine signaling coexists with poor CD8^+^ T-cell infiltration or myeloid-enriched inflammation.

Multi-pathway cooperative intervention represents a further direction for therapeutic development. Metabolism, inflammation, and stromal remodeling are highly coupled processes in PDAC. Extracellular purine metabolism can regulate immune cell recruitment and activation (Ma Z. et al., 2026), whereas lipid metabolism and related signal transduction pathways can jointly promote immune escape (Lao et al., 2025; Jiang et al., 2026). At the same time, targeting stromal cells or fibrotic structures, such as specific CAF subsets, can relieve barriers to immune cell entry into the tumor core and restore antitumor immune responses (Krishnamurty et al., 2022). For myeloid-rich tumors, combining myeloid trafficking blockade with T-cell activation may provide a direct therapeutic strategy. In PDAC models, simultaneous targeting of CXCR1/2, LAG3, and 4-1BB reduces suppressive myeloid constraints and enhances T-cell function, resulting in durable antitumor response (Gulhati et al., 2023). Therefore, simultaneous intervention at multiple levels, including metabolism, inflammation, CAF-ECM barriers, and immune checkpoints, may more effectively reshape the TME. However, such strategies still face challenges related to heterogeneity and safety. PDAC exhibits marked heterogeneity, and different KRAS mutation subtypes and microenvironmental states may correspond to distinct metabolic dependencies and immune features (Ardalan et al., 2025). In addition, many metabolic pathways also have fundamental functions in normal immune cells, and broad inhibition may cause immune impairment or systemic toxicity. Therefore, future studies should combine multi-omics analyses with functional validation to identify selective key nodes at different disease stages. Overall, combination intervention based on cytokine–metabolic crosstalk aims to systematically target multiple key regulatory nodes, thereby breaking immune exclusion and restoring effective antitumor immune responses.

### 5.3 Key therapeutic targets in cytokine–metabolic crosstalk

During cytokine–metabolic crosstalk, some molecules are not restricted to a single pathway but instead connect inflammatory signaling, metabolic reprogramming, and immune suppression, making them potential therapeutic targets for combination strategies. STAT3, NF-κB, and HIF-1α are representative integrative nodes. TNF-related inflammatory signaling can activate the STAT3 pathway, drive cholesterol metabolic reprogramming, and promote tumor progression, suggesting that STAT3 serves as a key bridge between inflammation and metabolism (Jiang et al., 2026). NF-κB not only regulates inflammatory factor expression but also participates in metabolism-related gene transcription and myeloid cell recruitment, thereby maintaining an immunosuppressive microenvironment (Liu et al., 2023; Chen Q et al., 2025). Under hypoxic and metabolic stress conditions, HIF-1α can simultaneously regulate glycolysis and inflammation-related signaling, reinforcing metabolic adaptation and immune suppression in tumor cells (Sun et al., 2025).

In addition to classical signaling nodes, metabolites and inflammatory factors may also function as actionable links. Lactate and related metabolic enzymes can promote the formation of an immunosuppressive microenvironment and enhance immune escape through signaling axes such as STAT3/CCL2 (Sun et al., 2025). IL-6 and its downstream signaling may form a feedback-like regulatory process between tumor cells and CAFs, contributing to tumor progression and chemoresistance (Hu et al., 2022). TAMs can further reinforce microenvironmental remodeling and malignant progression by secreting multiple cytokines and interacting with tumor cells (Liu et al., 2024). Therefore, identifying these connecting nodes from the perspective of a cooperative network may help overcome the limitations of single-pathway targeting and provide a more rational basis for developing targeted combination therapeutic strategies.

### 5.4 Biomarkers and patient stratification

The immunosuppressive state of PDAC shows marked spatial and molecular heterogeneity, and different patients vary substantially in TME composition, cellular functional states, and therapeutic responses. Therefore, patient stratification is essential. Single-cell transcriptomic and spatial omics studies have shown that PDAC progression is accompanied by dynamic changes in cellular subpopulations and the immune microenvironment, and different molecular subtypes vary in the degree of immunosuppression and therapeutic response (Park et al., 2024; Chen M. M. et al., 2025). The infiltration levels of specific immune cell subsets may also have stratification value. For example, CD161^+^CD8^+^ T cells are associated with immunosuppressive phenotypes and prognosis, making them candidate biomarkers of immune status (Chen et al., 2024). Genetic driver events further shape stratification features. KRAS wild-type and KRAS-mutant tumors display distinct immune infiltration patterns and molecular characteristics, suggesting that genotype may influence sensitivity to immunotherapy (Philip et al., 2022). Beyond this distinction, different KRAS alleles may exhibit distinct biological characteristics. For example, KRAS^G12R^ differs from KRAS^G12D^ and KRAS^G12V^ in PI3K pathway engagement and macropinocytosis, suggesting that KRAS allele type should be considered an important stratification factor when evaluating molecular features, metabolic dependency, and therapeutic response (Hobbs et al., 2020; Ardalan et al., 2025). Tumor cell plasticity also contributes to therapeutic heterogeneity; ROR2 signaling can regulate tumor cell state transitions and KRAS-dependent pathway activity, thereby influencing tumor progression and therapeutic responses (Benitz et al., 2024).

Tumor antigenicity represents another important determinant of immunotherapy response (McGranahan et al., 2016; Yarchoan et al., 2017). Most PDAC tumors exhibit relatively low tumor mutational burden (TMB) and limited neoantigen generation, which may reduce tumor immunogenicity and limit cytotoxic T-cell recognition, contributing to the limited efficacy of immune checkpoint blockade (Waddell et al., 2015; Balachandran et al., 2017; Bear et al., 2020). In contrast, the microsatellite instability-high/deficient mismatch repair (MSI-H/dMMR) subgroup represents a rare molecular context characterized by increased mutational burden, enhanced neoantigen generation, and greater sensitivity to immunotherapy (Humphris et al., 2017; Le et al., 2017; Coston et al., 2023). However, immune responsiveness in PDAC is likely determined by the interaction between tumor antigenicity and the immunosuppressive microenvironment, and the relative contributions of insufficient antigenicity *versus* immune exclusion remain incompletely defined.

Metabolic and cytokine-related features provide additional dimensions for stratification. Indoleamine 2,3-dioxygenase 1 (IDO1)-mediated tryptophan metabolism and nicotinamide adenine dinucleotide (NAD)-related pathways can regulate the immunosuppressive state and participate in the transition between “cold” and “hot” tumor phenotypes (Anu et al., 2023). CAFs also influence the microenvironment by secreting cytokines and participating in vasculogenic mimicry and stromal remodeling, and different CAF subtypes are associated with prognosis and therapeutic responses (Biffi et al., 2019; Elyada et al., 2019; McAndrews et al., 2022). Therefore, patient stratification in PDAC should not rely on a single biomarker, but should integrate spatial architecture, KRAS genotype, immune cell composition, metabolic status, and CAF features. Establishing a multidimensional “genotype–immune–metabolic” biomarker system may help identify the key network dependencies of different patients and provide a basis for individualized combination therapeutic strategies.

### 5.5 Challenges and future directions

Combination strategies targeting cytokine–metabolic interactions have a solid biological rationale, but their clinical translation still faces several challenges. Multi-pathway inhibition may increase toxicity risk because many metabolic and inflammatory pathways are also required for normal immune cell function and tissue homeostasis. Broad inhibition of these pathways may therefore impair antitumor immunity or cause systemic adverse effects. Patient heterogeneity further complicates clinical application. The TME of PDAC differs markedly across patients in immune cell composition, stromal density, spatial architecture, metabolic dependency, and dominant immunosuppressive mechanisms, making it possible for the same treatment strategy to produce divergent therapeutic responses in different patients (Ho et al., 2020).

Current biomarkers remain insufficient to effectively distinguish tumors mainly driven by cytokine signaling from those dominated by metabolic suppression, stromal barriers, or mixed regulatory mechanisms. Most combination regimens are still at the preclinical or early exploratory stage, and the optimal drug combinations, treatment sequence, dose intensity, and patient selection criteria remain to be defined. Future clinical development should prioritize biomarker-guided stratification strategies that integrate KRAS genotype, immune phenotype, metabolic state, and stromal features, rather than broad, non-selective combination therapy.

## 6 Research gaps and future perspectives

Several scientific questions remain unresolved. KRAS is the predominant driver gene in PDAC and can link tumor-cell-intrinsic programs with stromal and immune-cell states by regulating cytokine production and metabolic remodeling (Dey et al., 2020). However, how KRAS-driven cytokine–metabolic crosstalk is organized *in vivo* and how it maintains immune exclusion remain unclear. Most studies have focused on individual pathways, metabolic processes, or specific cell populations, leaving the integrated organization of the native tumor ecosystem insufficiently understood.

The spatial and temporal organization of this network also requires further study. Single-cell and spatial multi-omics studies have shown that CAFs and other stromal components occupy specific tissue regions and interact with tumor and immune cells (Liu et al., 2025c). However, the major sites of cytokine–metabolic crosstalk remain undefined. These interactions may be enriched at tumor–stroma interfaces, hypoxic myeloid-rich niches, invasive fronts, or regions with dense CAF/ECM deposition. At these sites, IL-6, CXCLs, TNF-α, and TGF-β may cooperate with lactate accumulation, extracellular acidity, adenosine signaling, and nutrient restriction to shape CD8^+^ T-cell exclusion, suppression, or entry into tumor nests. The immunosuppressive state of PDAC also changes during tumor progression and treatment, making it difficult to determine which factors initiate immune exclusion, how tumor-cell-intrinsic KRAS programs, stromal barrier formation, myeloid-cell accumulation, and metabolic stress are ordered, and whether this hierarchy varies across tumor states.

Future studies should shift from describing individual pathways to defining which cytokine–metabolic interactions are causal for immune exclusion. Integrating spatial omics, metabolic flux analysis, organoid–immune–stromal co-culture systems, and *in vivo* perturbation models may help identify causal cytokine–metabolic circuits and determine how these interactions vary across tumor states and treatment conditions. These studies may clarify how KRAS mutation shapes the immunosuppressive microenvironment beyond tumor proliferation and help identify key network nodes for reversing immune exclusion in PDAC.

## 7 Conclusion

KRAS-driven cytokine signaling and metabolic reprogramming are closely connected in PDAC. KRAS-related secretory programs promote myeloid cell recruitment, CAF activation, and stromal remodeling, whereas metabolic changes, including glycolytic activation, lactate accumulation, acidic TME formation, and adenosine signaling, further suppress antitumor immunity. Shared nodes such as STAT3, NF-κB, HIF-1α, and MYC link these processes and allow cytokine signals and metabolic stress to reinforce each other within the TME.

This cytokine–metabolic crosstalk helps explain key features of immune exclusion in PDAC, including myeloid cell enrichment, CAF/ECM-associated barriers, restricted CD8^+^ T-cell infiltration, and poor response to immunotherapy. Immune exclusion is therefore unlikely to be maintained by a single pathway. Instead, it reflects coordinated changes in cytokine signaling, metabolism, stromal remodeling, and immune-cell distribution. In this context, KRAS mutation should be considered not only as a tumor cell-intrinsic oncogenic event, but also as a genetic background that shapes the immunosuppressive TME. A clearer understanding of this network may help identify more rational targets for disrupting immune exclusion and improving immunotherapy responses in PDAC.
